# The p97 Inhibitor UPCDC-30245 Blocks Endo-Lysosomal Degradation

**DOI:** 10.3390/ph15020204

**Published:** 2022-02-07

**Authors:** Feng Wang, Shan Li, Kai-Wen Cheng, William M. Rosencrans, Tsui-Fen Chou

**Affiliations:** 1 Division of Biology and Biological Engineering, California Institute of Technology, Pasadena, CA 91125, USA; lflshan@caltech.edu (S.L.); kwcheng@caltech.edu (K.-W.C.); wrosencr@caltech.edu (W.M.R.); 2 Proteome Exploration Laboratory, Beckman Institute, California Institute of Technology, Pasadena, CA 91125, USA

**Keywords:** proteomics, p97 inhibitor, lysomotropic agents, endo-lysosomal degradation, coronavirus

## Abstract

The diverse modes of action of small molecule inhibitors provide versatile tools to investigate basic biology and develop therapeutics. However, it remains a challenging task to evaluate their exact mechanisms of action. We identified two classes of inhibitors for the p97 ATPase: ATP competitive and allosteric. We showed that the allosteric p97 inhibitor, UPCDC-30245, does not affect two well-known cellular functions of p97, endoplasmic-reticulum-associated protein degradation and the unfolded protein response pathway; instead, it strongly increases the lipidated form of microtubule-associated proteins 1A/1B light chain 3B (LC3-II), suggesting an alteration of autophagic pathways. To evaluate the molecular mechanism, we performed proteomic analysis of UPCDC-30245 treated cells. Our results revealed that UPCDC-30245 blocks endo-lysosomal degradation by inhibiting the formation of early endosome and reducing the acidity of the lysosome, an effect not observed with the potent p97 inhibitor CB-5083. This unique effect allows us to demonstrate UPCDC-30245 exhibits antiviral effects against coronavirus by blocking viral entry.

## 1. Introduction

Developing p97 inhibitors is an emerging strategy to treat cancers by targeting protein quality control (PQC) pathways [1,2,3]. Two well-known p97 inhibitors, CB-5083 and NMS-873, induce apoptosis in a number of cancer cell lines via impairment of endoplasmic-reticulum-associated protein degradation (ERAD) and activation of the unfolded Protein response pathway (UPR) [1,2,3,4]. UPCDC-30245, a representative of phenyl indole-based allosteric p97 inhibitors, is a potent inhibitor of p97 ATPase activity [3,5]. Physiologically, UPCDC-30245 has been demonstrated to suppress cell proliferation, with IC_50_ in the nanomolar to low micromolar range [3]. UPCDC-30245 represents a promising alternative against p97-mutant cancers that are resistant to ATP-competitive p97 inhibitors such as CB-5083 [3]. However, the cellular effects of UPCDC-30245 on ERAD, UPR, and autophagy are distinct from CB-5083 and another type of allosteric p97 inhibitor, NMS-873. While the treatments with CB-5083 and NMS-873 lead to the accumulation of ubiquitinated proteins, activation of UPR, and the reduction of autophagy adaptor p62 [1,2,3,4], UPCDC-30245 displayed only weak effects on protein ubiquitination, UPR, and p62, but strongly increased LC3-II level in HCT116 cells [3].

p97 controls multiple cellular functions via the formation of multiprotein complexes with diverse adaptor proteins, also known as p97 cofactors [6]. The varying cellular responses to different p97 inhibitors may stem from specific effects on p97 complexes [7,8]. The unique performance of UPCDC-30245 may be the result of interfering with distinct p97 complexes. Whether the unique action of UPCDC-30245 is a consequence of p97-complex alteration or an off-target effect is not yet known.

Mass spectrometry-based proteomics has been widely used in the field of drug discovery and development, for the discovery of new molecules as therapeutic targets [9,10,11,12] and validating the mechanism of action (MOA) of known compounds [13,14,15]. We recently compared temporal proteomic profiling to compare three p97 inhibitors (CB-5083, NMS-873 and UPCDC-3025) with shRNA knockdowns of p97 [16] and we observed different pathways are affected by UPCDC-3025. In this study, we perform additional label free mass spectrometry based proteomic analysis on HCT116 cells treated with UPCDC-30245 or DMSO. Through the identification of proteomic changes and dysregulated cellular functions, we aim to uncover the unique MOA of UPCDC-30245 and its potential applications. We found that UPCDC-30245 dysregulates endo-lysosomal degradation. p97 does play critical role in endosomal pathways such as interacting with early endosomal autoantigen 1 (EEA1) to regulate size of early endosome [17], regulated endosomal sorting of cavelolin-1 [18], and is required to clear ruptured lysosomes by autophagy [19]. The endolysosome pathway consists of endocytosis, formation of early endosome (pH 5.5–6.0), late endosomes (pH 5.5–5.0), and lysosomes (pH 5.0–4.5) and a fusion lysosomes and late endosomes to form endolysosomes.

## 2. Results

### 2.1. UPCDC-30245 Displays Unique Cellular Effects Compared to Two Other p97 Inhibitors

**Figure 1:**
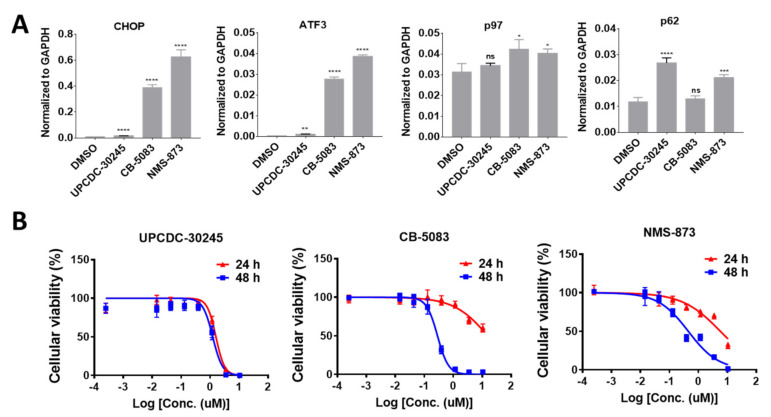
Differential cellular effects between UPCDC-30245 and CB-5083 or NMS-873 in HCT116 cells. (**A**) qRT-PCR analysis of CHOP, ATF3, p97 and p62 RNAs following treatment with DMSO, 5 μM of UPCDC-30245, 5 μM of CB-5083 or 5 μM of NMS-874 for 6 h. *N* = 3. ns, not significant; *, *p* < 0.05; **, *p* < 0.01; ***, *p* < 0.0005; ****, *p* < 0.0001; according to one-way ANOVA with multiple comparison tests. (**B**) The cellular viability curves for UPCDC-30245, CB-5083 and NMS-873. *N* = 4.

In our previous study, UPCDC-30245 displayed a mild effect on the protein levels of UPR activated genes (CHOP and ATF4) in HCT116 cells, distinct from the action of CB-5083 and NMS-873 [3]. In HCT116 cells, UPCDC-30245 upregulates the transcription of CHOP and ATF3 by 20-fold less than that of CB-5083 and NMS-873 (Figure 1A). Unlike CB-5083 and NMS-873, UPCDC-30245 did not affect the expression of p97 but significantly upregulated the expression of p62 by 2.6-fold (Figure 1A). In addition, UPCDC-30245 stopped HCT116 cells proliferation beginning at 24 h after treatment. This result suggests its anti-proliferative effect is more acute than CB-5083 and NMS-873 (Figure 1B). These data demonstrate that the cellular effects of UPCDC-30245 are distinct from that of CB-5083 and NMS-873.

### 2.2. Proteomics Reveals Impairment of Endo-Lysosomal Pathways in Cells Treated with UPCDC-30245

**Figure 2:**
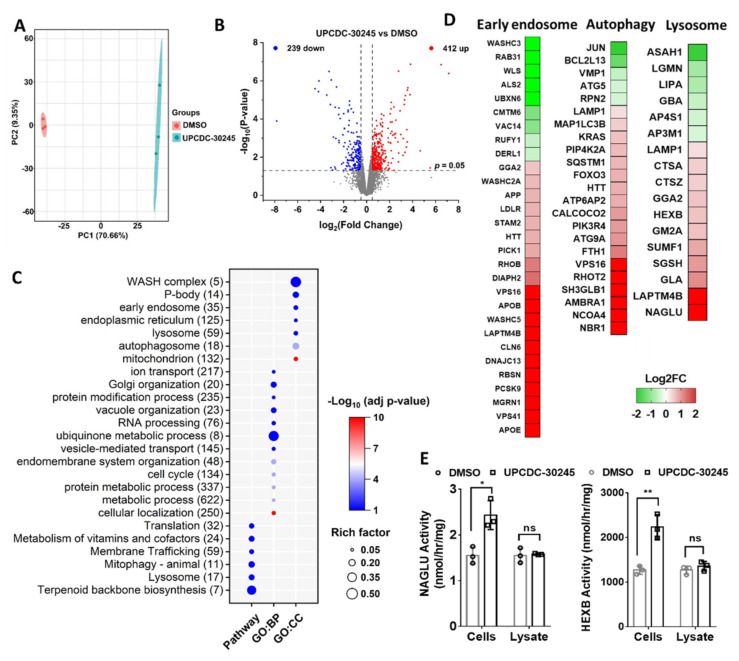
Proteomics reveals impairment of endo-lysosomal pathways in cells treated with UPCDC-30245 in HCT116 cells. (**A**) PCA revealed a separation between the treatment with DMSO and 5 of UPCDC-30245 for 6 h. (**B**) Volcano plot displaying the proteomic changes following UPCDC-30245 treatment in HCT116 cells, log_2_ (fold change) indicates the logarithm to the base 2 of fold change, *n* = 3. (**C**) Functional enrichment analysis on proteins affected by UPCDC-30245. (**D**) Heatmap showing fold change in early endosome proteins, autophagy and lysosome related proteins which are significantly dysregulated by UPCDC-30245. (**E**) Enzymatic activity of HEXB and NAGLU. Cells indicates enzymatic activity was detected in HCT116 cells pre-treated with DMSO or 5 μM of UPCDC-30245 for 6 h. Lysate indicates enzymatic activity was detected using by treating HCT116 cell lysate with DMSO or 5 μM UPCDC-30245 for 30 min. An increase in enzyme activity was observed in the cells treated with UPCDC-30245, but not in the lysate, which indicates that UPCDC-30245 increased the amount of enzyme. *N* = 3. *, *p* < 0.05; **, *p* < 0.01; according to one-way ANOVA tests.

Our recent proteomic study has identified a set of proteins which were dysregulated by p97 knockdown and p97 inhibitors, CB-5083 and NMS-873 [16]. Those proteins were linked to pathways typically affected by p97 inhibition, such as protein processing in the ER, UPR, and asparagine N-linked glycosylation. Interestingly, UPCDC-30245 had no effect on the majority of these proteins [16]. Which indicates that compared with CB-5083 and NMS-873, UPCDC-30245 has a unique MOA. Functional enrichment analysis revealed that the proteins dysregulated by the treatment with UPCDC-30245 for 6 h were linked to autophagy pathway (Figure S1A). To increase the number of biological replicates, we preformed proteomic analysis of HCT116 cells treated with DMSO or UPCDC-30245 for 6 h in triplicate using label-free quantification. Samples were analyzed via a 2 h gradient run on nano-LC coupled with Eclipse mass spectrometry. We identified a total of 7286 proteins from all 6 samples. After excluding the proteins with two missing values in both groups, 6272 remained. Principal component analysis (PCA) showed a separation between the treatments along principal component 1 (Figure 2A). Differential expression (DE) analysis was performed on the 6272 proteins by Limma [20]. We identified 900 proteins that demonstrated significantly different (*p* < 0.05) quantities between the samples treated with DMSO or UPCDC-30245. Of the 900 differential expressed proteins (DEPs), 412 were increased by UPCDC-30245 with log_2_ fold change >0.5 and 239 were decreased with log_2_ fold change < −0.5 (Figure 2B).

To reveal the cellular functions that are potentially affected by UPCDC-30245, we performed functional enrichment analysis on the 900 DEPs using g:Profiler website (https://biit.cs.ut.ee/gprofiler/gost, accessed on 16 January 2022). As shown in Figure 2C, the DEPs are linked to diverse cellular functions, including metabolism, RNA processing, membrane trafficking, autophagy and lysosome.

From terms, including early endosome (GO:CC), Mitophagy (KEGG), autophagosome (GO:GC) and lysosome (KEGG), which are involved in lysosomal degradation, we have identified 59 early endosome components, 23 and 17 DEPs, which are linked to autophagy and lysosome, respectively. Interestingly, the levels of most DEPs linked to early endosome, autophagy and lysosome were elevated by UPCDC-30245, including lysosome components (LAMP1, NAGLU and HEXB) and proteins that are normally degraded by lysosome via autophagy (amyloid precursor protein-APP) [21] or recycled via endocytosis (LDLR, APOB and APOE) [22] (Figure 2D). These data indicate that UPCDC-30245 may dysregulate endo-lysosomal degradation. In addition, the increases in LDLR, APOB and NAGLU were also observed from our previous proteomic data (Figure S1B) [16]. To validate our proteomic data, we used enzymatic activity assays to measure the active enzyme levels of NAGLU and HEXB [23,24]. Our results showed UPCDC-30245 treated samples have significant increase of HEXB and NAGLU enzymatic activities (Figure 2E), and we demonstrated that UPCDC-30245 did not affect their activities when added directly to cell lysate (Figure 2E), which is consistent with the increased protein levels in our proteomic data.

### 2.3. UPCDC-30245 Inhibits the Formation of Early Endosome and Autophagy Flux

**Figure 3:**
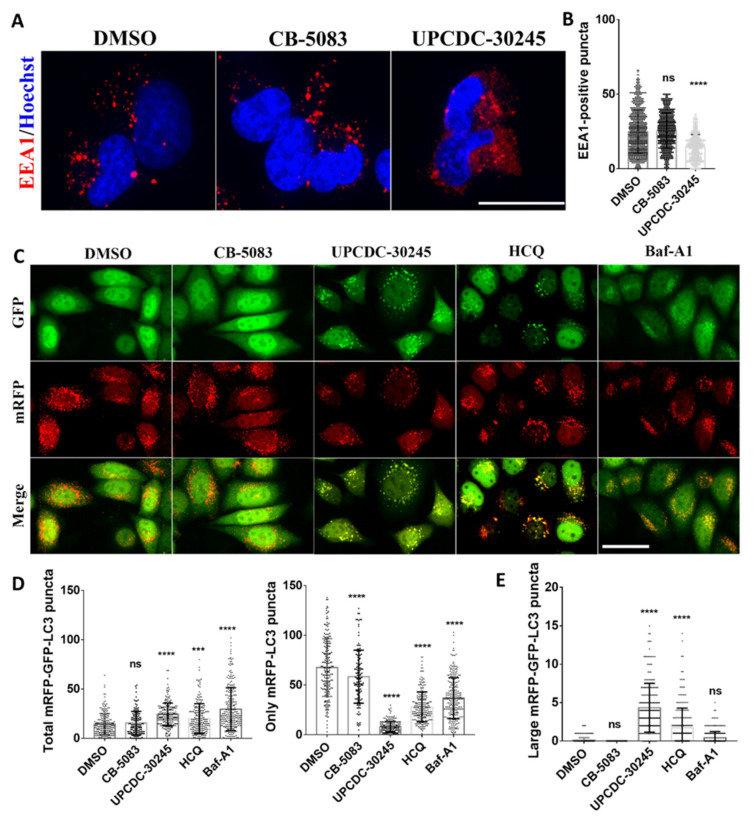
UPCDC-30245 inhibits the formation of early endosome and autophagy flux in H1299 and HeLa cells. (**A**,**B**) UPCDC-30245 (5 μM) inhibits the formation of EEA1 positive puncta after 1 h of treatment in H1299. 550 (DMSO), 528 (CB-5083) and 228 (UPCDC-30245) cells were counted from triplicates. Scale bar represents 25 μm. (**C**,**D**) The number of autophagosomes were increased and autolysosomes were reduced by 5 μM of UPCDC-30245, 50 μM of HCQ and 10 μM of Baf-A1, but not by 5 μM of CB-5083 in HeLa cells stably expressing mRFP-GFP-LC3 reporter after 2 h of treatment. Scale bar represents 50 μm. (**E**) UPCDC-30245 and HCQ induced the formation of large autophagosomes in HeLa cells after 2 h of treatment. The 210 (DMSO), 158 (CB-5083), 215 (UPCDC-30245), 225 (HCQ) and 291 (Baf-A1) cells were counted from four replicates. ns, not significant; ***, *p* < 0.0005; ****, *p* < 0.0001 according to one-way ANOVA with multiple comparison tests.

Functional enrichment analysis on the DEPs showed that UPCDC-30245 potentially dysregulates endocytosis and autophagy. UPCDC-30245 increases multiple protein levels belongs to early endosome components (LDLR, APOB and APOE) (Figure 2D). To evaluate the effects of UPCDC-30245 on endocytosis, we assessed the early endosomes by observing the early endosome marker EEA1 [25,26]. To clearly detect and quantify early endosomes (EEA1-positive vesicles), we performed immunofluorescence using H1299 cells instead of HCT116 cells, which are flatter and attach better after multiple washes. In the presence of UPCDC-30245, the occurrence of early endosomes was decreased significantly in the cytosol (Figure 3A,B); however, the protein level of EEA1 was not reduced by the treatment with UPCDC-30245 (Table S1). Instead of forming puncta, EEA1 distributed throughout the cytosol in cells treated with UPCDC-30245 (Figure 3A). These effects were not observed in cells treated with CB-5083 (Figure 3A). Our data suggest that UPCDC-30245 may block proper formation of the early endosome.

To evaluate the effect of UPCDC-30245 on autophagy, we employed a tandem mRFP-GFP-LC3 fluorescence reporter, which is widely used to assess autophagy flux, in HeLa cells [27]. This autophagy biosensor utilizes GFP fluorescence which rapidly quenched in acidic compartments whereas RFP fluorescence is more stable. Increasing levels of autophagy correspond to elevated levels of RFP-only (red) puncta. As expected, both hydroxychloroquine (HCQ) and bafilomycin-A1 (Baf-A1) treatments significantly elevated the number of autophagosomes (both GFP and mRFP positive puncta) and reduced the formation of autolysosomes (only mRFP positive puncta) (Figure 3C,D). UPCDC-30245 exhibited a significant increase in the total number of autophagosomes and a dramatic reduction of autolysosomes (Figure 3C,D). CB-5083 displayed no significant effect on the autophagosomes while slightly reduced autolysosomes. Measuring the size of these puncta, we observed that UPCDC-30245 and HCQ induced formation of larger puncta than that induced by Baf-A1. These data suggested that UPCDC-30245 and HCQ blocks autophagy in a similar fashion.

### 2.4. UPCDC-30245 Directly Disturbs the Acidic Environment of Lysosomes

**Figure 4:**
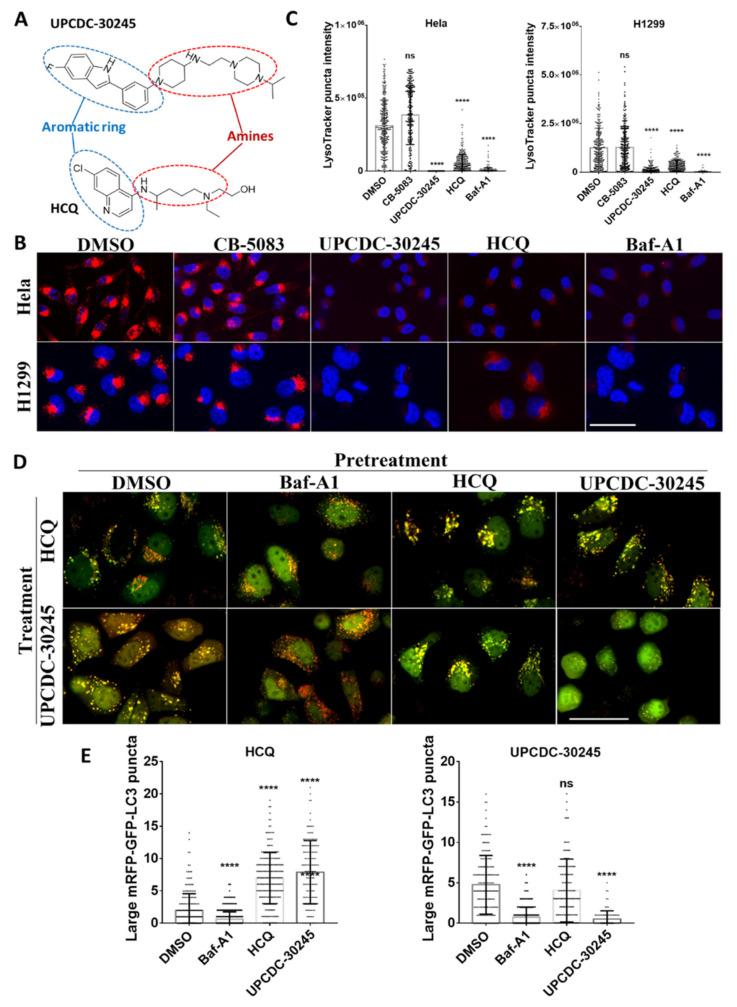
UPCDC-30245 decreases the acidity of lysosomes in H1299 and HeLa cells. (**A**) Structures of UPCDC-30245 and HCQ. (**B**,**C**) UPCDC-30245 (5 μM) decreases the LysoTracker puncta staining in both HeLa and H1299 cells. 220 to 290 (H1299) and 250 to 350 (HeLa) were counted from triplicates. Scale bar represents 50 μm. (**D**,**E**) Decreasing the acidity of lysosomes using Baf-A1 blocks the formation of large autophagosomes induced by UPCDC-30245 and HCQ in HeLa cells. HeLa cells were pretreated with DMSO, 10 μM of Baf-A1, 50 μM of HCQ or 5 μM of UPCDC-30245 for 30 min. Then 5 μM of UPCDC-30245 or 50 μM of HCQ were added. Images were taken at 2 h after the addition of UPCDC-30245 and HCQ. A total of 141 to 201 (HCQ) and 81 to 161 (UPCDC-30245) cells were counted from triplicates. Scale bar represents 50 μm. ns, not significant; ****, *p* < 0.0001 according to one-way ANOVA with multiple comparison tests.

Both UPCDC-30245 and HCQ are amphipathic molecules consisting of an aromatic ring and amine group (Figure 4A). The similar morphological features of their autophagy inhibition prompted us to hypothesize that their common structural feature could enable UPCDC-30245 to recapitulate the effects of HCQ that lead to lysosomal alkalinization [28]. To test our hypothesis, we measured the acidic organelles at 2 h after treatment using LysoTracker Red DND-99 (LysoTracker). LysoTracker is a pH-sensitive lysosomal dye, widely used to monitor the acidity of lysosomes [29]. The assay was performed on two different cell lines, HeLa and H1299, to exclude cell-specific effects. As both are known to alkalinize the lysosomes, the treatment with Baf-A1 and HCQ decreased LysoTracker puncta staining, as expected (Figure 4A,B). The decrease of LysoTracker puncta staining was also observed in the treatment with UPCDC-30245 but not CB-5083 (Figure 4A,B). This observation suggests that the decrease of LysoTracker puncta staining induced by UPCDC-30245 is a unique effect independent of general p97 inhibition. To exclude the possibility that UPCDC-30245 directly interfered with the lysosomal localization of LysoTracker, we stained the H1299 cells with LysoTracker prior to treatment with compounds and then assessed the acidity of lysosomes over time (Figure S2). UPCDC-30245 significantly decreased the LysoTracker puncta staining from 10 to 30 min after treatment. Therefore, our data suggest that UPCDC-30245 directly disturbs the acidic environment of lysosomes.

HCQ increases lysosome pH due to the accumulation of its protonated form in lysosomes [28] while Baf-A1, an inhibitor of vacuolar-type proton-ATPase (V-ATPase) also known as the vacuolar proton pump [30], prevents the luminal acidification of lysosomes. The lysosomal accumulation of HCQ leads to the formation of large autophagosomes. Protonation of the basic residues of HCQ is essential for its lysosomal retention (lysosomal trapping) only occurs in acidic lysosomes [28]. When we pre-treated HeLa cell with Baf-A1 for 30 min to decrease the acidity of lysosomes, both HCQ and UPCDC-30245 lost the ability to induce the formation of large autophagosomes (Figure 4D,E). In contrast, cells treated with UPCDC-30245 following pretreatment with HCQ did not show reduction in the formation of large autophagosomes, and vice versa (Figure 4D,E). Cells treated with two doses of UPCDC-30245 rounded up and no longer formed large autophagosomes at 2 h after the 2nd dose of UPCDC-30245. These data further confirm that HCQ and UPCDC-30245 share the same MOA by potentially increasing the pH of lysosomes and resulting in dysregulated autophagy.

### 2.5. UPCDC-30245 Inhibits Coronavirus Infection at Viral Entry Stage

**Figure 5:**
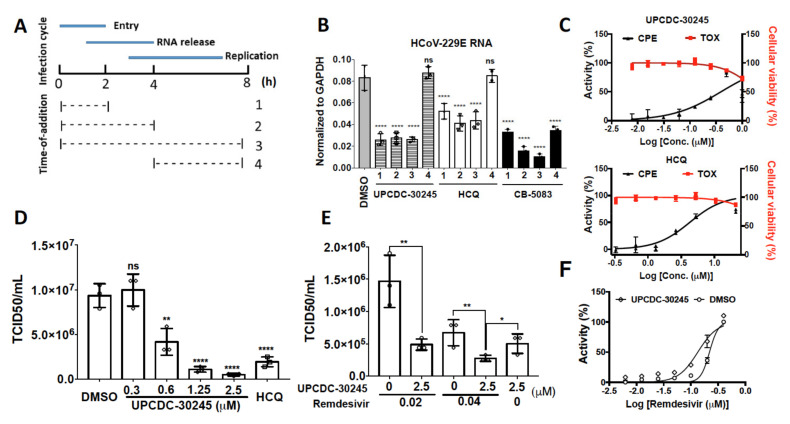
UPCDC-30245 inhibits HCoV-229E infection via blocking virus entry in H1299 cells. (**A**) Schema of time-of-addition experiment. 1. Added compound for 5 min, added virus for 2 h, washed out, added fresh media with DMSO for 6 h. 2. Added compound and virus together for 2 h, washed out, added fresh media with compound for 2 h, washout, add fresh media and DMSO for 4 h. 3. Added compound and virus together for 2 h, washed out, added fresh media with compound for 6 h. 4. Added DMSO and virus together for 2 h, washed out, added fresh media with DMSO for 2 h, washed out, and added fresh media with compound for 4 h. (**B**) The HCoV-229E RNA levels in H1299 cells were significantly reduced by 5 μM of UPCDC-30245 and 50 μM of HCQ at 8 h post infection. Data were normalized to the RNA levels of human GAPDH. Numbers 1 to 4 represent the time-of-addition described in Figure 5A. *N* = 3. (**C**) The protection of CPE activity (black curve) and toxicity (TOX, red curve) curves for UPCDC-30245 and HCQ. *N* = 4. (**D**) TCID_50_/mL measurements in a viral titer reduction assay for DMSO, UPCDC-30245 and 25 μM of HCQ after 24 h of HCoV-229E infection. *N* = 3. (**E**) TCID_50_/mL measurements for remdesivir. DMSO or 2.5 μM of UPCDC-3245 was added 5 min before virus infection and washed out with virus after 4 h of infection, then remdesivir was added and incubated for 20 h. *N* = 3. (**F**) The protection of CPE activity curves for remdesivir (0–0.42 μM, 3-fold dilution) with or without the pretreatment of 2.5 μM of UPCDC-30245 during virus infection. *N* = 3. ns, not significant; *, *p* < 0.05; **, *p* < 0.01; ****, *p* < 0.0001 according to one-way ANOVA with multiple comparison tests.

Since UPCDC-30245 can affect both endosome and autophagy functions and the effect on lysosome is similar to HCQ, we sought to determine whether UPCDC-30245 could affect human coronavirus (HCoV) infection in cell models as observed for HCQ [31,32,33]. We measured the HCoV-229E RNA levels in the host cytoplasm in a time-of-addition experiment for both HCQ, UPCDC-30245 and CB-5083 [34]. HCoV-229E is one of the seven HCoVs known to cause human infection [35]. In this experiment, virus was added at 0 h and washed out at 2 h and compounds were added at different time points (Figure 5A). Both HCQ and UPCDC-30245 significantly decreased HCoV-229E RNA levels when they were added 5 min before the virus infection, incubated with virus for 2 h, and washed out (1, 0 to 2 h) and reached plateau following an additional 2 h treatment post infection (2, 0 to 4 h) (Figure 5B). The extended UPCDC-30245 treatment duration up to 8 h (3, 0 to 8 h) did not further reduce viral RNA load. Both HCQ and UPCDC-30245 were unable to reduce the virus RNA levels when they were added 2 h post infection (4, 4 to 8 h). These results indicate that UPCDC-30245 blocks HCoVs entry to the host cells and release of the viral RNA genome into the host cytoplasm, but has no direct effect on viral replication, consistent with its ability to simultaneously inhibit the formation of early endosome and lysosomal degradation. Consistent with the effects of UPCDC-30245, 5 μM of CB-5083 significantly decreased in HCoV-229E RNA levels when CB-5083 was added during the virus infection (1, 0 to 2 h) (Figure 5B). However, the longer treatment duration (2, 0 to 4 h or 3, 0 to 8 h) led to further decrease of the HCoV-229E RNA into cytosol. In addition, CB-5083 reduced virus RNA levels when it was added at 2 h post infection (4, 4 to 8 h). These data demonstrate that CB-5083 had strong effects on virus entry, release of the viral RNA genome, and virus replication.

To explore whether UPCDC-30245 has an anti-viral effect, we employed a cell-based assay that measures the cytopathic effect (CPE) caused by HCoV-229E as described previously [36]. In this assay, cell viability is used to measure the protection effect of potential antiviral therapeutics on host-cell death due to viral infection and we recently identified HCoV-229E caused strong CPE in H1299 and showed some representative CPE images after viral infection [37]. After 6 days of treatment, both HCQ and UPCDC-30245 inhibited the CPE of HCoV-229E with EC_50_ of 4.2 and 0.4 μM, respectively (Figure 5C). However, significant cytotoxicity was observed at 1 μM of UPCDC-30245 and 42 μM of HCQ (Figure 5C). The effect of UPCDC-30245 on viral titer was measured by the median tissue culture infectious dose (TCID_50_). To avoid cytotoxicity, UPCDC-30245 and HCQ were added 5 min before virus infection and washed out with virus after 4 h of infection. UPCDC-30245 concentration-dependently reduced the viral titer at 20 h post infection. The minimum effective concentration was found to be 0.6 μM (Figure 5D). When remdesivir, an inhibitor of RNA-dependent RNA polymerase for coronavirus [38], was added after washout of UPCDC-30245, the virus titer displayed a further reduction (Figure 5E). In addition, the treatment with UPCDC-30245 during virus infection increased the activity of remdesivir in inhibiting CPE of HCoV-229E (Figure 5F).

## 3. Discussion

Development of p97 inhibitors is an alternative strategy to treat cancers by targeting PQC pathways. The potent ATP-competitive active site inhibitors, CB-5083 and CB-5339, displayed efficacy in mouse xenograft models implanted with HCT116 cells [1,39]. The phase I clinical trials of CB-5083 in patients with multiple myeloma and advanced solid tumors were halted due to off-target retinal toxicity related to inhibition of PDE6 [40]. CB-5339, a new compound was developed to avoid the PDF6 off-target effect [39]. Recently, NMS-873, a potent allosteric p97 inhibitor, was reported to inhibit mitochondrial oxidative phosphorylation, due to its off-target effect [41]. As a representative of the 2nd class of allosteric p97 inhibitors, UPCDC-30245 displayed distinct cellular effects comparing to CB-5083 and NMS-873 [3]. Therefore, uncovering the MOA of UPCDC-30245 is critical for the p97 research community.

In this study, we identified the proteomic changes in HCT116 cells caused by UPCDC-30245 and revealed the cellular functions which are altered by UPCDC-30245 (Figure 2A,C). In addition to the role in ERAD, p97 is also implicated in membrane trafficking [42,43], mitochondrial dynamics [44], Golgi and ER biogenesis [45,46] and mRNA stability [47]. It is thus not surprising that processes associated with p97 functions, including vesicle-mediated transport, Golgi organization, mRNA processing, endoplasmic reticulum, and mitochondrion, were enriched from the proteins dysregulated by UPCDC-30245 (Figure 2C). These results suggest that UPCDC-30245 affects the cellular functions of p97. However, no process associated with ubiquitin-mediated proteolysis and UPR, the most well-known hallmarks of p97 inhibition, was identified. Similarly, our previous study revealed UPCDC-30245 was unable to cause the accumulation of ubiquitinated proteins and activate UPR [3]. The generation of drug resistant cell line is a powerful tool to study the on-target or off-target effects. However, after several attempts we were unable to develop a UPCDC-30245 resistant cell line. By contrast, we were able to generate several CB-5083 and NMS-873 resistant cell lines. There are several possible explanations for this difference: (1) UPCDC-30245 inhibits specific p97 dependent endosome and autophagy functions more potently than it does ERAD and UPR function. (2) UPCDC-30245 inhibits specific p97 complexes in cells that are different from CB-5083 and NMS-873. (3) UPCDC-30245 has off-target effects and future studies are needed to identify the hypothetical off-target.

From our proteomics data, UPCDC-30245 dysregulates early endosome and lysosome components, and affects lysosome, endocytosis and autophagy related pathways (Figure 2C,D). By assessing EEA1-positive vesicles, we found UPCDC-30245 reduces the formation of early endosomes (Figure 3A). p97 is reported to be involved in endocytosis [17,48], this phenomenon was not observed in the cells treated with CB-5083. Therefore, the reduction of early endosomes is a cellular effect potentially unique to UPCDC-30245. The upregulation of LAMP1, lysosomal enzymes (HEXB and NAGLU) as well as autophagy has been found in multiple lysosome storage diseases (LSDs) [23,24,49]. LSDs are caused by the insufficient degradation of (GAGs) due the deficiency of certain lysosome enzymes. Agents that inhibit lysosomal functions can act as mimetics of acute LSDs [50]. Of those agents, HCQ inhibits lysosomal functions via increasing lysosome pH [28]. Through a tandem mRFP-GFP-LC3 fluorescence analysis, we found UPCDC-30245 and HCQ performed similarly in inhibiting autophagy flux and inducing the formation of large autophagosomes (Figure 3B). The reduction in LysoTracker staining confirmed that UPCDC-30245 reduces the acidity of lysosomes (Figure 4B, Figure S1). Interestingly, we observed the formation of large autophagosomes in the cells treated with HCQ and UPCDC-30245 rather than the cells treated with Baf-A1 (Figure 3B). When the cells were pretreated with Baf-A1 to reduce the acidity of lysosomes, HCQ and UPCDC-30245 were unable to induce the formation of large autophagosomes (Figure 4D), indicating that the formation of large autophagosomes could be a consequence of lysomotropic agents. In addition, these data confirm UPCDC-30245 functions as a lysomotropic agent that directly inhibits lysosomal functions.

Recent studies have documented that p97 is an essential host protein used by viruses to infect host cells and that inhibition of p97 exhibited anti-viral activity against influenza as well as SARS-CoV-2 [34,51,52,53,54]. HCQ/CQ and other small molecules which alkalize lysosomes displayed antiviral effects against human coronavirus (HCoV) [31,55,56,57]. UPCDC-30245 displays the ready features of a promising antivirus agent as it simultaneously inhibits p97, lysosomal degradation and the formation of early endosomes. Through a time-of–addition experiment, we confirmed that UPCDC-30245 blocks the host-cell entry and genome RNA release of HCoV-229E (Figure 5B) and that CB-5083 blocks multiple steps during HCoV-229E infection (Figure 5B). It is possible that UPCDC-30245 inhibits specific p97 dependent functions that are different from those inhibited by CB-5083. In addition, UPCDC-30245 inhibits the CPE of HCoV-229E and reduces the virus titer (Figure 5C,D). Moreover, pretreatment with UPCDC-30245 elevates the antivirus activity of remdesivir (Figure 5E,F).

## 4. Material and Methods

### 4.1. Cell Culture

HCT116 and H1299 cells were grown in RPMI1640 medium (Corning, 10-040-CM), HeLa cells were grown in DMEM medium (Sigma, D5796-1L), and cultured at 37 °C in a 5% CO_2_ incubator. Culture medium was supplemented with 10% FBS (R&D, S11150) and 1% penicillin-streptomycin (Gibco, 15140-122).

### 4.2. RNA Extraction and qPCR Analysis

Cells were harvested and pellets were re-suspended in DPBS/TRIzol-LS mixture (Ambion, 10296010; *v*/*v* 1:3). Total RNA was extracted from the TRIzol-LS mixture using Direct-zol RNA MiniPrep plus kit (Zymo Research, Irvine, CA, USA, R2072) according to the manufacturer’s instructions. Purified RNA was converted to cDNA using SensiFAST™ cDNA Synthesis Kit (Meridian Bioscience, Cincinnati, OH, USA, BIO-65054). Quantitative PCR (qPCR) was performed on the QuantStudio™ 5 Real-Time PCR System (Thermo Scientific, Waltham, MA, USA, A28140) using TaqMan Universal Master Mix II no UNG (Thermo Scientific, 4440040). 2ˆ(-∆CT) values were calculated after normalizing to GAPDH levels. A list of the qPCR probes used is provided in Supplementary Table S1.

### 4.3. Anti-Proliferative Activity

Anti-proliferative activity was measured using the Cell Titer Glo^®^ Luminescent Cell Viability Assay (Promega G7572) according to the manufacturer’s procedure. HCT116 cells were re-suspended in RMPI1640 containing 5% FBS and 1% penicillin-streptomycin. Cells were seeded at 30 μL cell suspension (750 cells) per well in 384 well plates (Greiner 781080). On the second day, 8 μL of assay media containing compounds or 5% DMSO was added into each well, and the plates were incubated for an additional 24 or 48 h at 37 °C in a 5% CO_2_ incubator. Cell viability was measured by CellTiter Glo, and IC50 values were calculated using the percentage of growth of treated cells versus the DMSO control. The results were analyzed using GraphPad Prism 7.0.

### 4.4. Immunofluorescence Staining

H1299 cells were seeded at 20,000 cells per well in a 96 well plate (Greiner, 655090). On the second day, cells were treated with DMSO or compounds for 1 h. Then, cells were fixed with 4% PFA and blocked with 10% donkey serum and 0.1% Triton X-100 in 1x DPBS for 1 h. After overnight incubation with anti-EEA1 (Abcam, ab2900, 1:50) at 4 °C, Alexa Fluor 594-conjugated secondary antibody (Invitrogen, A-21207, 1:500) was applied to cells. Nuclei were stained with Hoechst33342 (Thermo Scientific, 62249, 1 ug/mL). Images were taken and analyzed using ImageXpress^®^ Confocal HT.ai High-Content Imaging System (Molecular Devices, San Jose, CA, USA).

### 4.5. Enzymatic Activity Assays for NAGLU and HEXB

NAGLU activity was determined using the NAGLU substrate, 4-methylumbelliferyl 2-acetamido-2-deoxy-α-D-glucopyranoside (Toronto Research Chemicals, North York, ON, Canada, M333800) and HEXB activity was determined using the HEXB substrate, 4-methylumbelliferyl-N-acetyl-β-glucosaminide (EMD Millipore Chemicals, 474502). For NAGLU activity, reaction mixtures consisted of 2.5 μL of cell lysates, 2.5 μL of 4 mM NAGLU substrate in 200 mM acetate buffer pH 5.6 with 0.01% Triton. For HEXB activity, cell lysate was diluted 1000-fold using PAD buffer (10 mM sodium phosphate, pH 5.8, 0.02% sodium azide, 0.1 mM dithiothreitol, 0.1% Triton X-100), reaction mixtures consisted of 2.5 μL of cell lysates and 2.5 μL of 2.5 mM HEXB substrate in PAD buffer. After incubation for 2 h at 37 °C, the mixtures were quenched by adding 65 mL of 500 mM glycine/sodium carbonate pH 10.6. The reaction mixtures were transferred into a 384 well plate. Fluorescence measurements were obtained using a SpectraMax^®^ iD5 Multi-Mode Microplate Reader (Molecular Devices, San Jose, CA, USA) at excitation and emission wavelengths of 360 nm and 450 nm, respectively.

### 4.6. Tandem mRFP-GFP-LC3 Fluorescence Assay

HeLa cells stably expressing mRFP-GFP-LC3 reporter were plated in a 96 well plate (Greiner, Kremsmünster, Austria, 655090) and grown for 18 h. After 2 h of treatments with DMSO or compounds, images were taken using ImageXpress^®^ Confocal High-Content Imaging System (Molecular Devices). Experiments were performed in 3 wells and images were taken from 9 sites per well. The puncta/cell was counted from all the 27 images and used to perform statistical analysis. Total cell numbers were indicated in the figure ligand. The quantification was performed using custom model of MetaXpress 6 Software (Molecular Devices). The co-localization (overlay) of GFP puncta and RFP puncta was considered as autophagesome. The criteria for large puncta is that the dot have a diameter larger than 2 μm, otherwise it will be considered as a small puncta.

### 4.7. Measuring the Cytotoxicity and Anti-CPE Effects of Compounds

H1299 cells were re-suspended in RPMI1640 medium supplemented with 2% FBS and 1% P/S. A total of 20 μL of cell suspension containing 1250 cells was plated in 384 well plates and incubated for 18 h at 35 °C in a 5% CO_2_ incubator. Compounds were serially diluted 2-fold and added to cell plates. After 2 h of treatment, 10 uL of HCoV-229E was added to cell plates (MOI = 0.1) for anti-CPE assay or fresh assay medium was added for cytoxicity assay. Then, the plates were incubated at 35 °C for 6 days. Cell viability was measured by CellTiter Glo. Raw data from each test well were normalized to the average reading of the noninfected cells (avg. mock; 100% inhibition) and virus-infected cells only (avg. virus; 0% inhibition) to calculate anti-CPE activity using the following formula: % activity = 100 × (test cmpd − avg. virus)/(avg. mock − avg. virus).

### 4.8. Time-of Addition-Assay

H1299 cells were re-suspended in RPMI1640 medium supplemented with 2% FBS and 1% P/S and plated at 0.5 × 10^6 cells per well in 6 well plates. After incubating for 18 h at 35 °C in a 5% CO_2_ incubator, cells were infected with HCoV-229E (MOI = 1) and treated with compounds at different time points. Virus were washed at 2 h post infection and cells were harvested at 8 h post infection.

### 4.9. TCID50 Assay

H1299 were plated same as described in the time-of-addition assay. Compounds were added 5 min before virus infection and washed out with virus at 4 h post infection. Culture medium containing secreted virus was collected at 24 h post infection. For TCID50 measurement, H1299 were plated same as described in CPE assay. After overnight incubation, the 10-fold serially diluted culture medium was added to cell plates. After 6 days, cell viability was measured by CellTiter Glo. TCID50 was calculated using the Reed and Muench method [58].

### 4.10. Label-Free Proteomics

Samples were prepared using the Thermo EasyPep Mini MS Sample Prep Kit (Thermo Scientific, A4006) according to the manufacturer’s instructions. The dried peptides were then dissolved in 0.1% formic acid (Thermo Scientific, 5178) solution, and peptide concentration was tested using the Pierce Quantitative Fluorometric Peptide Assay (Thermo Scientific, 23290). LC-MS/MS experiments were performed on an EASY-nLC 1000 (Thermo Fisher Scientific, Waltham, MA, USA) connected to an Orbitrap Eclipse Tribrid mass spectrometer (Thermo Fisher Scientific). Proteomic analysis was performed using Proteome Discoverer 2.4 (Thermo Scientific) software, the Uniprot human database and SequestHT with Percolator validation. The detailed information for LC/MS processing and data analysis was described previously [59].

## 5. Conclusions

Taken together, we found that UPCDC-30245 dysregulates endo-lysosome degradation pathways through proteomic analysis, and validated that UPCDC-30245 inhibits the formation of early endosomes. We also revealed the unique effect of UPCDC-30245 in blocking autophagy flux due to UPCDC-30245′s ability to directly alkalinize lysosomes. The effect on lysosomal pH and the inhibition of p97 caused by UPCDC-30245 leads to inhibition of HCoVs host cell entry and genomic RNA release into the cytosol suggests its potential as an antiviral agent.

## Data Availability

All relevant data generated during this study are included in the article and the supplementary Information. The mass spectrometry raw data are deposited to the ProteomeXchance Consortium (https://www.ebi.ac.uk/pride/, accessed on 28 December 2021) via the PRIDE repository with the dataset identifier PXD025822 and 10.6019/PXD025822”. Additional raw data generated during the current study and relevant information are available from the corresponding authors upon request. The data are not publicly available due to the larger size and complexity.

## Supplementary Materials

The following are available online at https://www.mdpi.com/article/10.3390/ph15020204/s1, Figure S1: (A) Functional enrichment analysis on proteins affected by UPCDC-30245. (B) The log2 fold change of LDLR, APOB and NAGLU from previous and new data set, Figure S2: UPCDC-30245 rapidly reduced the LysoTracker staining in H1299 cells. Table S1: qPCR probes used in this study.
