# Supplementary material for: The p97 Inhibitor UPCDC-30245 Blocks Endo-Lysosomal Degradation
Source: Pharmaceuticals (Basel). 2022 Feb 7;15(2):204. doi: 10.3390/ph15020204 (PMC8880557; doi:10.3390/ph15020204)
Supplement: Supplementary file 1 [file pharmaceuticals-15-00204-s001.zip › pharmaceuticals-1562477-supplementary.pdf]

Supplementary information

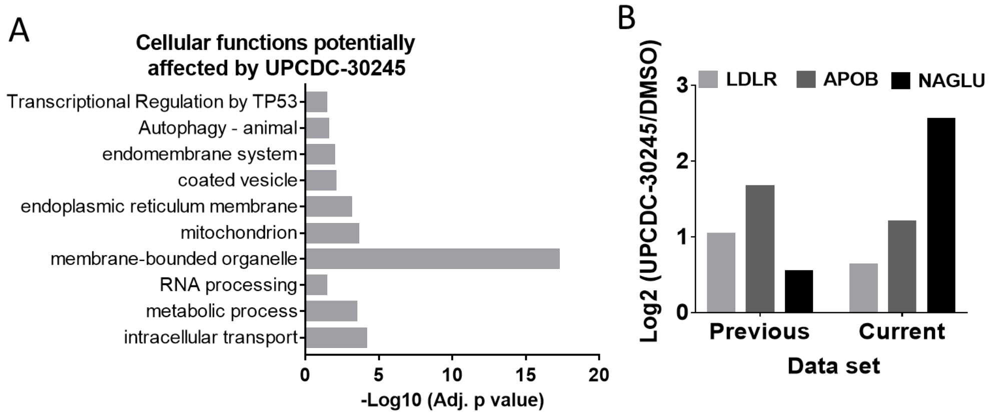

Figure S1. (**A**) Functional enrichment analysis on proteins affected by UPCDC-30245. HCT116 cells were treated with 4 μM UPCDC-30245 for 6 hours then label free proteomic was performed on the prepared samples, *n* = 2. DE analysis was performed using Proteome Discoverer 2.4 [1] and re-analyzed using gprofiler (https://biit.cs.ut.ee/gprofiler/gost). (**B**) The log2 fold change of LDLR, APOB and NAGLU from previous [1] and new data set.

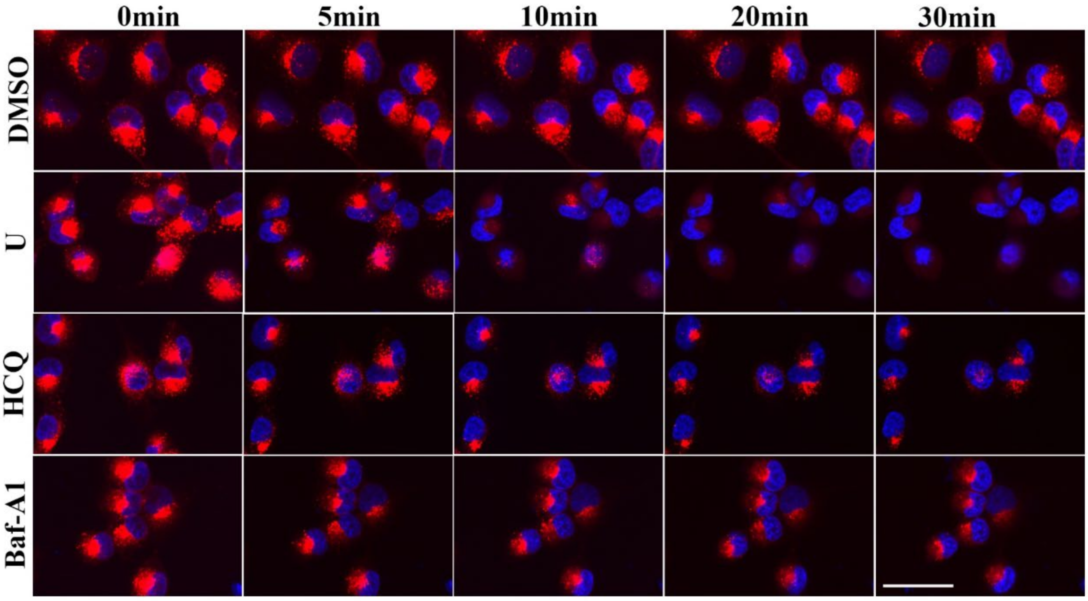

Figure S2. UPCDC-30245 rapidly reduced the LysoTracker staining in H1299 cells. Cells were pre-treated with LysoTracker for 30 min. After removing LysoTracker, DMSO, 5 μM of UPCDC-30245 (U), 50 μM of HCQ or 10 μM of Baf_A1 were added to the cells. Images were taken at 0, 5, 10, 20 and 30 min after the addition of compounds. Scale bar indicates 50 μm.

Table S1 qPCR probes used in this study

Target	Catalog Number
GAPDH	Hs02786624_g1
HCoV-229E	Vi06439671_s1
p62	Hs00177654_m1
p97	Hs00997640_g1
ATF3	Hs00231069_m1
CHOP	Hs01090850_m1

Reference

1. Wang, F., et al., *Temporal proteomics reveal specific cell cycle oncoprotein downregulation by p97/VCP inhibition.* Cell Chem Biol, 2021.
